# Obesity, diet quality and absenteeism in a working population

**DOI:** 10.1017/S1368980016001269

**Published:** 2016-05-27

**Authors:** Sarah Fitzgerald, Ann Kirby, Aileen Murphy, Fiona Geaney

**Affiliations:** 1 Department of Epidemiology and Public Health, University College Cork, 4th Floor, Western Gateway Building, Western Road, Cork, Republic of Ireland; 2 School of Economics, University College Cork, Cork, Republic of Ireland

**Keywords:** Absenteeism, Obesity, Diet quality, Zero-inflated binomial regression, Workplace dietary intervention

## Abstract

**Objective:**

The relationship between workplace absenteeism and adverse lifestyle factors (smoking, physical inactivity and poor dietary patterns) remains ambiguous. Reliance on self-reported absenteeism and obesity measures may contribute to this uncertainty. Using objective absenteeism and health status measures, the present study aimed to investigate what health status outcomes and lifestyle factors influence workplace absenteeism.

**Design:**

Cross-sectional data were obtained from a complex workplace dietary intervention trial, the Food Choice at Work Study.

**Setting:**

Four multinational manufacturing workplaces in Cork, Republic of Ireland.

**Subjects:**

Participants included 540 randomly selected employees from the four workplaces. Annual count absenteeism data were collected. Physical assessments included objective health status measures (BMI, midway waist circumference and blood pressure). FFQ measured diet quality from which DASH (Dietary Approaches to Stop Hypertension) scores were constructed. A zero-inflated negative binomial (zinb) regression model examined associations between health status outcomes, lifestyle characteristics and absenteeism.

**Results:**

The mean number of absences was 2·5 (sd 4·5) d. After controlling for sociodemographic and lifestyle characteristics, the zinb model indicated that absenteeism was positively associated with central obesity, increasing expected absence rate by 72 %. Consuming a high-quality diet and engaging in moderate levels of physical activity were negatively associated with absenteeism and reduced expected frequency by 50 % and 36 %, respectively. Being in a managerial/supervisory position also reduced expected frequency by 50 %.

**Conclusions:**

To reduce absenteeism, workplace health promotion policies should incorporate recommendations designed to prevent and manage excess weight, improve diet quality and increase physical activity levels of employees.

The growing prevalence and associated burden of chronic diet-related diseases is endangering population health and the sustainability of health-care systems worldwide^(^ ^1^ ^,^ ^2^ ^)^. Obesity and obesity-related diseases including CVD, stroke and diabetes have been linked to absenteeism and productivity loss in the workplace, generating substantial costs for societies and employers^(^ ^3^ ^)^. In 2011 it was reported that absenteeism due to illness was costing Irish businesses €1·5 billion per year (€818 per employee per year)^(^ ^4^ ^)^. Furthermore, productivity loss due to overweight and obesity was estimated at €865 million in 2009 in Ireland, with absenteeism identified as the main driver^(^ ^5^ ^)^. In an effort to curtail this cost escalation, workplace health promotion has moved to the forefront of organisational agendas. However, it is imperative that workplace health promotion guidelines and polices are developed and informed by objective research^(^ ^6^ ^)^.

The workplace has been identified as a priority setting for health promotion as it can facilitate the delivery of health promotion initiatives by providing necessary infrastructure and a stable population in a controlled environment^(^ ^1^ ^,^ ^2^ ^,^ ^7^ ^,^ ^8^ ^)^. Given that employees are spending longer periods of time in their work environments, the workplace has the capacity to influence the physical, mental, economic and social well-being of employees and consequently the health and well-being of their families and communities^(^ ^6^ ^,^ ^8^ ^)^. It is widely accepted that health promotion should form a fundamental element of workplace culture as the future success of organisations in a progressively globalised marketplace is dependent on establishing a balance between organisational targets and employees’ health^(^ ^8^ ^)^.

In terms of health status outcomes, much research into the determinants of workplace absenteeism has focused on obesity. The most recent available data for Ireland suggest that overweight and obesity rates may have reached a plateau in Irish adults but at a high level, with 61 % of adults being classified as overweight (37 %) and obese (24 %)^(^ ^9^ ^)^. Obesity has been identified as a significant predictor of sick leave and studies indicate that a gradient exists between obesity and absenteeism^(^ ^10^ ^–^ ^15^ ^)^. This gradient may be attributable to the adverse health implications associated with obesity, which increase the likelihood of absences occurring^(^ ^3^ ^,^ ^12^ ^)^. There is consensus in the evidence base regarding the relationship between obesity and absenteeism; however, a limitation of the evidence is the wide use of self-reported measures for absenteeism and obesity^(^ ^13^ ^,^ ^14^ ^)^. Moreover, recent evidence suggests that general obesity measures such as BMI can lead to misclassification of body fat^(^ ^13^ ^,^ ^16^ ^)^. Central obesity is a strong indicator of chronic diet-related diseases and, thus, a potential predictor of sick leave^(^ ^13^ ^,^ ^16^ ^)^. However, further research is warranted as few studies have included both measures of central obesity and BMI^(^ ^13^ ^)^.

The causes of workplace absenteeism are multifaceted and are not limited to obesity and health status outcomes. Absenteeism may be attributable to many different factors, including lifestyle factors, demographic and socio-economic characteristics. The relationship between absenteeism and modifiable lifestyle characteristics remains ambiguous. For example, both positive^(^ ^12^ ^,^ ^17^ ^)^ and negative^(^ ^11^ ^)^ associations between physical activity and absenteeism have been reported. Similarly, inconsistent findings have been reported regarding smoking status and absenteeism^(^ ^11^ ^,^ ^12^ ^,^ ^18^ ^,^ ^19^ ^)^. This uncertainty may be due to different outcome measurements being used and issues including type of work and working conditions^(^ ^12^ ^,^ ^20^ ^)^. In addition, inconclusive findings have been reported with respect to the relationship between absenteeism and alcohol consumption^(^ ^21^ ^,^ ^22^ ^)^. In relation to diet quality, there is a paucity of evidence investigating the impact of dietary behaviours on absenteeism^(^ ^23^ ^)^. One of the few studies conducted reported that improvements in dietary behaviours such as reducing consumption of fatty foods and increasing fruit and vegetable intake significantly improved presenteeism of employees^(^ ^24^ ^)^. Owing to the aetiological role of diet in the causation and prevention of chronic diet-related diseases, it is vital that the impact of dietary behaviour on absenteeism is robustly examined^(^ ^25^ ^)^. With regard to demographic and socio-economic characteristics, studies have reported a clear negative linear association between socio-economic status and absenteeism^(^ ^26^ ^)^. Higher education level and increased job responsibility are associated with lower rates of absenteeism^(^ ^27^ ^,^ ^28^ ^)^.

The aim of the present study was to investigate what health status outcomes and lifestyle characteristics influence the frequency of workplace absenteeism, using objective measures for both absenteeism and health status outcomes. Findings will inform both employers and public health policy makers on what guidelines should be included in workplace health promotion policies in an effort to improve employee health and potentially reduce absenteeism.

## Methods

### Data source

Data for the present study were obtained from a large clustered controlled trial, the Food Choice at Work Study (FCW). Full details of the FCW protocol are published elsewhere^(^ ^29^ ^)^. The FCW assessed the comparative effectiveness of a workplace environmental dietary modification intervention and an educational intervention of high intensity both alone and in combination *v*. a control workplace. The present study is a cross-sectional analysis of baseline data collected prior to the implementation of the FCW interventions. The FCW had a sample size of 828 employees (aged 18–64 years). Employees were recruited from the four workplaces (*n* 100 (70 % response rate); *n* 224 (70 % response rate); *n* 392 (60 % response rate); *n* 112 (91 % response rate)). The number of employees recruited per workplace reflected the difference in company size^(^ ^29^ ^)^. Eligible employees were permanent, full-time employees who purchased and consumed at least one daily meal in work. Employees who were medically advised not to participate and those who were involved in an ongoing diet programme external to work were excluded from the study. Throughout the FCW, participants underwent physical assessments (height, weight, midway waist circumference and blood pressure) that were conducted by trained research assistants^(^ ^30^ ^)^. Demographic, nutrition knowledge and FFQ were self-completed by participants. Participants provided written informed consent to enable their workplaces to make their absence history available to the research team. A total of 540 employees consented, giving a response rate of 65 % for the present study.

### Absenteeism

Annual count absenteeism data for each participant were obtained from the Human Resources department of each workplace. Frequency of absences was recorded in working days, based on an 8 h working day (40 h working week). As this is a baseline cross-sectional analysis, absenteeism data were collected for a time period prior to the implementation of the FCW interventions (July 2012–July 2013). Maternity or paternity leave absences were excluded from the analysis. The total number of days absent during this time period was specified as the dependent integer count variable for analysis.

### Health status and lifestyle characteristics

Exposure data were collected at the baseline stage of data collection, prior to the implementation of the FCW interventions (February–July 2013). Measured BMI was calculated as kg/m^2^ in order to classify participants as underweight (BMI≤18·49 kg/m^2^), normal weight (BMI=18·50–24·99 kg/m^2^), overweight (BMI=25·00–29·99 kg/m^2^) or obese (BMI≥30·00 kg/m^2^)^(^ ^2^ ^)^. As BMI is a weight-for-height measure and is unable to distinguish between body fat and lean mass, midway waist circumference measurements (central obesity) were also included as an obesity indicator. Participants were classified as centrally obese if their midway waist circumference was measured at ≥94 cm for male or ≥80 cm for female participants^(^ ^31^ ^)^. Participants’ resting blood pressure was measured and participants were recorded as hypertensive if their average systolic reading was ≥140 mmHg or their average diastolic was ≥90 mmHg^(^ ^32^ ^)^.

An International Physical Activity Questionnaire (IPAQ) score was calculated for physical activity levels^(^ ^33^ ^)^. These scores were classified as low (<5000 steps/d), moderate (5000–10 000 steps/d) and high (>10 000 steps/d) levels of physical activity. Smoking status was recoded into three categories: ‘non-smokers’ (participants who had never smoked more than 100 cigarettes), ‘former smokers’ (participants who had smoked at least 100 cigarettes but do not smoke at present) and ‘current smokers’ (participants smoking at present)^(^ ^33^ ^)^. Alcohol consumption was estimated using the units of alcohol consumed per week.

Diet quality was assessed using an FFQ, which measured the average frequency of consumption of foods from nine food groups: whole grains, fruit, vegetables, legumes, low-fat dairy, red processed meat, sweetened snacks and beverages, salty snacks and sodium^(^ ^34^ ^)^. A DASH (Dietary Approaches to Stop Hypertension) score was constructed from the FFQ, with a high DASH score indicating high diet quality^(^ ^35^ ^)^. The DASH diet pattern promotes low intakes of fat, Na and processed foods and high intakes of fruit and vegetables^(^ ^35^ ^)^. This diet pattern has been found to lower blood pressure and cholesterol and is promoted internationally. To further assess Na intake, spot urine samples were obtained to analyse Na excretion. Each participant provided one early morning sample and one evening sample which were taken 12 h apart. Daily average salt intakes were estimated based on the average between both samples. These estimates were compared with the upper tolerable limit of 6 g/d for Irish populations as set out in national guidelines^(^ ^36^ ^)^.

Participants’ nutrition knowledge was assessed using the validated general nutrition knowledge questionnaire which included four sections: (i) advice from health experts; (ii) food groups and food sources; (iii) food choice; and (iv) diet–disease relationships. Nine questions were modified to include recent evidence in nutrition knowledge (e.g. ‘What health problems are related to excess sugar?’). Food items were changed to increase participants’ understanding (e.g. orange juice instead of orange squash). An overall score was constructed and further categorised into high or low nutrition knowledge score^(^ ^37^ ^)^. Sociodemographic and lifestyle characteristics (gender, age, ethnicity, marital status, education level, job position, smoking and physical activity level) were self-reported using a demographic questionnaire^(^ ^33^ ^)^. The highest level of completed education and job type served as indicators of socio-economic status. Education was transformed into a four-level variable: none/primary level only, secondary level only, diploma/certificate and degree/postgraduate level. Job type was also transformed into a four-level variable: human resources (HR)/finance/administration, information technology (IT)/engineering, production and maintenance/sanitation/catering. Job position was a measure of job responsibility and employees were classified as being in either a managerial/supervisory position or a non-managerial/non-supervisory position.

### Statistical analysis

All analyses were carried out using the statistical software package STATA version 12. Descriptive statistics were performed to generate a demographic profile of the study population’s baseline characteristics. As the annual number of days absent from work is a non-negative integer number, we employed a count data model to examine what health status outcomes and lifestyle factors influence the frequency of days absent from work. Previous studies have carried out multivariate analyses of count data models (Poisson, negative binomial and zero-inflated models) to establish what factors are associated with workplace absenteeism^(^ ^38^ ^,^ ^39^ ^)^. Typically, the Poisson model is the initial model considered when analysing count variables. Central to the Poisson model is the assumption that the conditional mean of the outcome is equal to the conditional variance. However, as is often the case, the conditional variance exceeds the mean, which results in overdispersion. In order to overcome the issue of overdispersion, negative binomial models can be used^(^ ^40^ ^)^. A zero-inflated negative binomial (zinb) model is employed when count variables have excessive zeros and are overdispersed. Moreover, a zinb model allows for excess zeros to be modelled independently^(^ ^41^ ^)^. In order to assess whether or not the zinb model was appropriate for our data, we applied a likelihood ratio test for *α*=0, the significance of which (*P*=0·0001) indicated the presence of overdispersion and preference for a zinb over a zero-inflated Poisson model (zip). We also applied the *vuong* non-nested model test statistic which compared the zinb with a negative binomial regression model and a significant *z*-test score (*z*=2·59, *P*>*z*=0·0048) indicated that the zinb was the best fit. Thus, a zinb was considered the most appropriate model due to overdispersion and the excessive frequency of zeros. We controlled for potential confounders including health (hypertension), lifestyle (smoking, alcohol consumption and nutrition knowledge) and sociodemographic (age, gender, education status and marital status) characteristics. Incident rate ratios were calculated for the zinb regression model and, in an effort to control for heterogeneity, robust standard errors were calculated.

## Results

With regard to absenteeism, 44 % of the study population (*n* 237) were absent at some stage during the specified period (July 2012–July 2013), with 14 % of employees absent for 1–2 d and 30 % of employees absent for 3 d or more. The mean number of absent days was 2·5 (sd 4·5) d. Baseline sociodemographic, lifestyle and physical characteristics for the study population are summarised in Table 1. The mean numbers of predicted days absent across different groups are also included in Table 1. The highest proportions of participants were male (67 %), aged 30–44 years (62 %), married/cohabiting (69 %) and were white Irish (90 %). A total of 42 % of the population had a tertiary education (degree or postgraduate degree level). Over 22 % of the population were in an HR, finance or administrative position, 29 % held an IT or engineering position and 39 % of the population worked in production. A total of 20 % of the population were in a managerial or supervisory position. A total of 18 % of the population were current smokers and 43 % reported having low levels of physical activity. A higher proportion of males (14 %) reported consuming at least 14 units of alcohol per week compared with females (3 %). Half of the participants were overweight (48 %) and centrally obese (51 %) and 15 % were hypertensive. According to urinary Na analyses, 39 % of the population exceeded the tolerable upper limit of 6 g salt/d. Over 60 % of participants had a low quality diet and 84 % had low levels of nutrition knowledge.

**Table 1:** Sociodemographic, health and lifestyle characteristics, by gender, of randomly selected employees from four multinational manufacturing workplaces in Cork, Republic of Ireland, February–July 2013 (Food Choice at Work Study)

	Men (*n* 359; 66·5 %)		Women (*n* 181; 33·5 %)		Total (*n* 540)		Mean number of
	*n*	%	*n*	%	*n*	%	predicted days absent
Sociodemographic characteristics							
Age group (years)							
18–29	37	10·3	25	13·8	62	11·5	2·8
30–44	226	63·0	106	58·6	332	61·5	2·7
45–65	96	26·7	50	27·6	146	27·0	2·1
Ethnicity							
White Irish	327	91·1	159	87·8	486	90·0	2·2
Other†	32	8·9	22	12·2	54	10·0	2·6
Educational level							
None/primary level only	5	1·4	1	0·6	6	1·1	2·8
Secondary level only	86	24·0	76	42·0	162	30·0	2·7
Diploma/certificate	92	25·6	52	28·7	144	26·7	2·8
Degree/postgraduate level	176	49·0	52	28·7	228	42·2	2·2
Marital status							
Married/cohabiting	267	74·4	104	57·5	371	68·7	2·5
Separated/divorced/widowed	14	3·9	13	7·2	27	5·0	2·2
Single/never married	78	21·7	64	35·3	142	26·3	2·7
Job type							
HR/finance/administration	64	17·8	57	31·5	121	22·4	2·4
IT/engineering	137	38·2	18	10·0	155	28·7	2·4
Production	115	32·0	95	52·5	210	38·9	2·8
Maintenance/sanitation/catering	43	12·0	11	6·0	54	10·0	2·3
Job position							
Manager/supervisor	88	24·5	20	11·0	108	20·0	1·3
Non-manager/non-supervisor	271	75·5	161	89·0	432	80·0	2·9
Health status outcomes							
BMI (kg/m^2^)‡							
Normal weight	80	22·3	76	42·0	156	28·9	2·3
Overweight	191	53·2	69	38·1	260	48·1	2·3
Obese	88	24·5	36	19·9	124	23·0	3·5
Central obesity§							
Normal	188	52·4	78	43·0	266	49·2	1·8
Centrally obese	171	47·6	103	57·0	274	50·8	3·2
Hypertension¶							
Not hypertensive	290	80·8	168	92·8	458	84·8	2·6
Hypertensive	69	19·2	13	7·2	82	15·2	2·3
Lifestyle characteristics							
Smoking status							
Never smoked	183	51·0	89	49·2	272	50·4	2·3
Former smoker	126	35·0	44	24·3	170	31·5	2·9
Current smoker	50	14·0	48	26·5	98	18·1	2·8
Alcohol consumption (units/week)							
No drink	75	20·9	54	29·8	129	23·9	2·8
1–<7	61	17·0	40	22·1	101	18·7	2·5
7–<14	48	13·4	16	8·8	64	11·9	2·2
14–<21/>21	49	13·6	5	2·8	54	10·0	2·4
Missing	126	35·0	66	36·5	192	35·5	
Physical activity							
Low	209	58·2	21	11·6	230	42·6	2·4
Moderate	78	22·6	76	42·0	154	28·5	1·8
High	69	19·2	82	45·3	151	28·0	3·5
Missing	3	0·8	2	1·1	5	0·9	
Daily salt intake							
≤6 g/d	208	58·0	121	66·9	329	61·0	2·5
>6 g/d	150	41·8	59	32·6	209	38·7	2·7
Missing	1	0·2	1	0·5	2	0·3	
DASH score (diet quality)							
High	112	31·2	92	50·9	204	37·8	1·9
Low	241	67·1	88	48·6	329	60·9	3·0
Missing	6	1·7	1	0·5	7	1·3	
Nutrition knowledge							
High	47	13·1	39	21·5	86	15·9	2·5
Low	312	86·9	142	78·5	454	84·1	2·7

HR, human resources; IT, information technology; DASH, Dietary Approaches to Stop Hypertension.

† Other: any other white, black or Asian ethnicities including mixed backgrounds.

‡ BMI: underweight, ≤18·49 kg/m^2^; normal weight, 18·50–24·99 kg/m^2^; overweight, 25·00–29·99 kg/m^2^; obese, ≥30·00 kg/m^2^.

§ Central obesity: midway waist circumference ≥94 cm for men or ≥80 cm for women.

¶ Hypertension: average systolic blood pressure ≥140 mmHg or average diastolic blood pressure ≥90 mmHg.

The variance inflation factor command was applied in order to check for the presence of multicollinearity between the diet quality, physical activity, BMI and central obesity variables. The variance inflation factor values computed were all less than 10, indicating the presence of minimal levels of correlation between the variables. We employed a zinb regression model to investigate what health status outcomes and lifestyle factors influence the frequency of workplace absences. The results of the zinb are provided in Table 2 and as the model includes a splitting function, the table is divided into two parts: the binary logit model and the negative binomial regression of the potential and actual number of absent days. The negative binomial regression generated statistically significant results for the variables job position, central obesity, physical activity and diet quality. Statistical significance was observed at the 5 % level of significance. The results indicate the presence of a negative relationship between the frequency of days absent and being in a managerial or supervisory position. This negative relationship was also reflected in the lower mean number of predicted days absent for managers/supervisors (1·3 d) *v*. non-managers/non-supervisors (2·9 d). A negative relationship was also observed between frequency of days absent and engaging in moderate levels of physical activity, which was again mirrored in the mean number of predicted days absent for physical activity levels (low, 2·4 d; moderate, 1·8 d; high, 3·5 d). Similarly, consuming a high-quality diet was negatively associated with frequency of absenteeism and the predicted number of days absent was lower for those consuming a high-quality diet (1·9 d) compared with those consuming a low-quality diet (3·0 d). A positive relationship was observed between frequency of days absent and being centrally obese. This positive relationship was replicated in the mean number of predicted days absent for this group, which was estimated to be 1·8 d for non-centrally obese employees and 3·2 d for centrally obese employees. No significant associations were found between absenteeism and age, education status, job type, marital status, BMI, daily Na intake, alcohol consumption, smoking status and hypertension.

**Table 2:** Zero-inflated negative binomial frequency of absent days among randomly selected employees (*n* 540) from four multinational manufacturing workplaces in Cork, Republic of Ireland, July 2012–July 2013 (Food Choice at Work Study)

	Logit model			Negative binomial model		
Variable	Coefficient	Robust se	*z* Statistic	IRR	Robust se	*z* Statistic
Job position	0·20	0·64	0·31	0·50**	0·11	−0·73
Central obesity	−0·91	1·12	−0·81	1·72**	0·49	1·91
Physical activity	−0·84	0·60	−1·41	0·50**	0·15	−2·30
BMI obese	−0·63	0·99	−0·64	0·91	0·27	−0·29
BMI overweight	0·40	0·96	0·42	0·88	0·21	−0·49
Nutrition knowledge	−1·50*	0·90	−1·67	0·99	0·29	−0·01
Diet quality	0·10	0·67	0·16	0·64**	0·12	−2·29
Constant	−0·33	2·67	−0·12	2·22**	0·72	3·09

IRR, incident rate ratio. **Indicates significance at the 5 % level, *indicates significance at the 10 % level.

The incident rate ratios calculated in the negative binomial part of the zinb model correspond to the coefficients for the percentage change in the expected count for participants who may have been absent from work (Table 3). Being in a managerial or supervisory position decreased the expected rate of absenteeism by 50 %. Similarly, regularly engaging in moderate levels of physical activity decreased the expected rate of absenteeism by 50 % and consuming a high-quality diet decreased the expected rate of absenteeism by 36 %. With respect to obesity, being centrally obese increased the expected rate of absenteeism by 72 %.

**Table 3:** Zero-inflated negative binomial model of percentage changes in coefficients and standard deviations of absent days among randomly selected employees (*n* 540) from four multinational manufacturing workplaces in Cork, Republic of Ireland, July 2012–July 2013 (Food Choice at Work Study)

	*b*	*z*	% Change	% StdX
Count equation: % change in expected count for those ‘not always 0’				
Job position	−0·68	−3·1	−49·5	−23·9
Central obesity	0·54	1·91	72·4	31·3
Physical activity	−0·69	−2·30	−50·0	−27·0
Diet quality	−0·44	−2·29	−35·5	−19·2
Binary equation: factor change in odds of ‘always 0’				
Job position	0·20	0·31	22·2	8·4
Central obesity	−0·91	−0·81	−59·8	−36·6
Physical activity	0·84	1·41	56·9	31·8
Diet quality	0·10	0·16	11·0	5·2

*b*, raw coefficient; *z*, *z* statistic score for test of *b*=0; % Change, percentage change in expected count for unit increase in *X*; % StdX, percentage change in expected count for sd increase in *X*.

The coefficients for the factor change in the odds of being in the ‘always zero’ group compared with the ‘not always zero’ group are also included in Table 3. Being in a managerial or supervisory position in work increased the odds of not being potentially absent from work by 22 %, holding all else constant. That is to say that the association between increased job responsibility and absenteeism remained, even after controlling for all other variables in the model. Being centrally obese decreased the odds of being present at work by 60 %. Consuming a high-quality diet increased the odds of not being absent from work by 11 % and engaging in moderate physical activity levels increased the odds of not being absent from work by 57 %. These associations persisted even after controlling for all other variables in the model.

## Discussion

The present study revealed four primary findings with regard to what health status outcomes and lifestyle factors influence the frequency of workplace absenteeism. Central obesity was found to significantly increase the expected frequency of absenteeism. With regard to lifestyle behaviours, consuming a high-quality diet decreased the expected frequency of absenteeism. Similarly, engaging in regular moderate physical activity also decreased the expected frequency of absenteeism. Furthermore, sociodemographic factors were also found to influence workplace absenteeism as increased job seniority reduced the expected frequency of absenteeism. Controlling for potential confounding sociodemographic, lifestyle and health characteristics did not alter these associations. The mean number of absent days was 2·5 (sd 4·5) d. In 2014, the Small Firms Association reported that for large businesses in Ireland, the average annual absenteeism rate was estimated to be 2·30 % (5·4 d) whereas in businesses with less than fifty employees, the rate was estimated to be 2·06 % (4·7 d)^(^ ^42^ ^)^. For these estimates, the Small Firms Association relied on self-reported estimates obtained from the Quarterly National Household Survey^(^ ^43^ ^)^. This difference in reporting method may explain the lack of concordance between the estimates as bias may have been introduced through self-reporting.

Obesity has been identified as a significant predictor of absenteeism in previous research^(^ ^10^ ^–^ ^15^ ^)^. Our overall findings support this consensus as central obesity was found to significantly increase the rate and potential occurrence of absenteeism. However, it is important to note that the results of the previous research are based on studies that relied on the use of both self-reported absenteeism data and self-reported BMI measurements whereas our study findings are based on objective measures for both absenteeism and obesity. In further contrast to previous research, we reported no significant findings between BMI and absenteeism as significance was observed only with the measurement of central obesity. As previously indicated, there is a growing body of evidence that suggests reliance on BMI for general obesity diagnosis can lead to misclassification of adiposity^(^ ^16^ ^)^. However, a dearth of evidence exists with regard to investigating the relationship between central obesity and absenteeism. In order to overcome issues with potential misclassification and to provide clarity on the appropriateness of obesity measures, future research should include robust objective BMI and midway waist circumference measurements. Consistency in future research will improve the comparability of our results.

Over 42 % of the study population was reported to have the highest level of educational attainment (degree or postgraduate degree). This is somewhat comparable to the national average of 34·3 %^(^ ^44^ ^)^. However, given that the participating workplaces were based in highly technical industries (automotive, medical devices, IT, and food and beverage), it could be argued that higher education levels among employees were to be expected. However, in contrast to previous literature, no association between educational attainment and absenteeism was reported^(^ ^26^ ^,^ ^27^ ^)^. Similarly, no association was found between job type and absenteeism. Our findings are consistent with current evidence that suggests increased job responsibility can influence workplace absenteeism and that greater decision authority is a predictor of lower absence rates^(^ ^27^ ^,^ ^28^ ^)^. Irrespective of job type, increased job responsibility and/or job seniority in the workplace may serve as a deterrent for high rates of absenteeism and also actual occurrence of absences.

As previously mentioned, a great deal of ambiguity exists in the evidence base with regard to the influence of physical activity on absenteeism with both positive and negative associations being reported^(^ ^11^ ^,^ ^12^ ^,^ ^17^ ^)^. Our findings of a negative association between absenteeism and physical activity may provide clarity to the evidence base due to the inclusion of objective absenteeism data. However, in order to accurately investigate the association between physical activity levels and absenteeism, the use of objective measures of physical activity through pedometers should be considered.

To date, no significant findings between absenteeism and diet quality have been reported^(^ ^24^ ^)^. A novel finding that has emerged from the present study is that consuming a high-quality diet (i.e. high fruit and vegetable consumption and low fat, sugar and salt consumption) can significantly reduce the frequency of absenteeism and also the potential for absences to occur. Workplace health promotion polices that include guidelines for creating a healthy eating environment may provide favourable return on investment for employers through reduced frequency of absent days^(^ ^45^ ^)^. Furthermore, three of the four principal findings influencing the frequency of absenteeism are modifiable health and lifestyle characteristics (i.e. obesity, diet quality and physical activity). This suggests that significant scope may exist to improve employee health outcomes and reduce absenteeism through the development of workplace health promotion policies. Such policies that are focused on increasing employees’ physical activity levels and improving their diet quality (increasing fruit and vegetable consumption and reducing intakes of fat, sugar and salt) should be critically considered by both employers and public health policy makers.

One of the key strengths of the current study is the use of recorded absenteeism data. Studies investigating predictors of absenteeism have relied heavily on self-reported absenteeism data. Using objective, recorded absenteeism considerably improves the quality and accuracy of the data and reduces the potential for measurement error, recall and social desirability bias. Similarly, objective measures for health status (BMI, central obesity and hypertension) were included and measured by trained research assistants. The four manufacturing workplaces involved in the study had similar structures and operations, ensuring employees had comparable demographics, health and lifestyle characteristics. There were very few missing data for the study, other than alcohol consumption. However, as these data were collected in the workplace, employees may have been reluctant to report their alcohol intake.

It is important to note a number of limitations of the current study. The FCW only included measures of physical health and occupational stress and other mental health indicators were omitted. Occupational stress has been highlighted as a significant contributor to workplace absenteeism^(^ ^46^ ^)^. The risk of obesity has been found to increase in work environments which are high-demand and low-autonomy^(^ ^28^ ^)^. Previous research has suggested that such environments can induce the occurrence of occupational stress in employees^(^ ^28^ ^,^ ^47^ ^)^. Stress has been found to negatively influence food choice in terms of saturated fat and sugar consumption, which can in turn lead to weight gain and subsequently absenteeism^(^ ^28^ ^)^. Future workplace health promotion studies should consider including measures of occupational stress alongside physical health outcome measures.

The sample size (*n* 540) could be interpreted as small; however, this is due to the exclusion criteria of the FCW trial. Exclusion criteria were focused on recruitment for the trial which may have influenced the findings of the present study. However, our study investigated the health status outcomes and lifestyle factors that influenced workplace absenteeism in a permanent manufacturing working population. To ensure there was an adequate response rate from employees per workplace, it was important that the study sample was contracted to work full-time and on a permanent basis in their workplaces. Furthermore, due to the cross-sectional design of the study, the findings should be interpreted cautiously due to the potential for reverse causality. However, the consistency between our results and published evidence regarding predictors of workplace absenteeism adds strength to our findings. Although it is very likely that the frequency of absenteeism is truly associated with central obesity, physical activity and diet quality, it is important to consider the potential for the presence of residual confounding in the data that was not captured or measured. Obesity is associated with a high number of adverse health implications which in turn increase the likelihood of absences occurring. It is possible that the association between central obesity and absenteeism may be driven by another factor arising from these adverse health implications that we have not considered in our data. An additional limitation to consider is with regard to hypertension. The effect of controlled hypertensives is unknown as medication data were unavailable. Additionally, we need to acknowledge the potential presence of the ‘healthy worker effect’. Despite employees being randomly selected to participate in the FCW, this bias cannot be ruled out as healthy employees may have been more likely to participate in the study, leading to potential underestimation of associations. It is also important to consider that measurement bias and social desirability bias may have been introduced to the data when estimating diet quality as the data were self-reported.

## Conclusions

In conclusion, the findings of the present study can be used to guide and inform the development of workplace health promotion guidelines and polices. Specifically, our results indicate that improving modifiable health and lifestyle characteristics including obesity, physical activity and diet quality should be at the core of such guidelines and policies to potentially reduce rates of absenteeism. Owing to the growing prevalence of obesity and its association with absenteeism, workplace health promotion policies should be focused on promoting strategies that can effectively prevent and reduce employees’ excess weight through increasing levels of physical activity and consuming a healthy diet. Implementation of informed workplace health promotion polices may benefit employers in terms of lowering rates of absenteeism and employees in terms of improved health outcomes.
